# Regenerative Therapies for Cosmetic Dermatology for Patients with Diabetes Mellitus: Skin Aging, Aesthetic Concerns, and Evidence-Based Best Practices

**DOI:** 10.3390/ijms27083507

**Published:** 2026-04-14

**Authors:** Tamara Tuma Odeh, Dillen A. Patel, Pradhyumna Mayur Pradeep, Jaiden A. Patel, Rahul Mittal, Khemraj Hirani

**Affiliations:** 1 Diabetes Research Institute, Miller School of Medicine, University of Miami, Miami, FL 33136, USA; tamara.tuma2000@gmail.com (T.T.O.); dillenpatel2008@gmail.com (D.A.P.); pradhyumnamayur.pradeep@gmail.com (P.M.P.); jxp2917@miami.edu (J.A.P.); 2 Monterrico Clinic, Avenida Manuel Olguín 1047, Santiago de Surco, Lima 15023, Peru; 3 Miami Herbert Business School, University of Miami, Miami, FL 33146, USA; 4 Division of Endocrinology, Diabetes, and Metabolism, Department of Medicine, Miller School of Medicine, University of Miami, Miami, FL 33136, USA

**Keywords:** diabetes mellitus, cosmetic dermatology, skin aging, advanced glycation end products, aesthetic procedures, regenerative medicine, platelet-rich plasma, platelet lysate, mesenchymal stromal cells, microneedling, best practices

## Abstract

Diabetes mellitus affects an estimated 589 million adults globally, and cutaneous manifestations occur in up to 70% of affected individuals during the course of the disease. The objective of this narrative review is to examine the intersection of diabetes mellitus, skin aging, cosmetic dermatologic procedures, and regenerative therapies, with an emphasis on evidence-based best practices and clinical considerations. While the impaired wound healing associated with diabetes has been extensively studied, the aesthetic implications of diabetic skin disease remain comparatively underexplored. Individuals with diabetes frequently exhibit features of accelerated cutaneous aging, including premature wrinkling, dyschromia, xerosis, alopecia, and other cosmetically significant dermatoses that may negatively impact quality of life. In parallel, the demand for aesthetic dermatologic procedures among patients with diabetes has increased substantially; however, evidence-based recommendations guiding the safe and effective use of cosmetic interventions in this population remain limited. Diabetic skin demonstrates accelerated biological aging driven by complex pathophysiological mechanisms, including the accumulation of advanced glycation end products, chronic low-grade inflammation, oxidative stress, microvascular dysfunction, and neuropathy. These processes partially overlap with chronological aging and photoaging but are mechanistically distinct and may influence tissue repair, inflammatory responses, and the safety profile of commonly performed aesthetic procedures such as chemical peels, laser resurfacing, dermal fillers, neuromodulators, and microneedling. Emerging regenerative approaches, including platelet-rich plasma, platelet lysate, and mesenchymal stromal cell-derived products such as exosomes and secretomes, have attracted increasing attention as biologically targeted strategies for cutaneous rejuvenation. Nevertheless, clinical evidence specifically addressing aesthetic interventions in diabetic populations remains limited. A diabetes-informed approach to aesthetic dermatology that considers metabolic status, procedure selection, and post-procedural monitoring is therefore essential to optimize safety and therapeutic outcomes.

## 1. Introduction

The intersection of diabetes mellitus and cosmetic dermatology represents a rapidly growing yet inadequately addressed clinical domain. With an estimated 589 million adults living with diabetes globally, a figure projected to reach 853 million by 2050 [1] and with cosmetic dermatology procedures exceeding 15 million annually in the United States alone [2], the population of patients seeking aesthetic care who also carry a diagnosis of diabetes is substantial and increasing.

**Figure 1:**
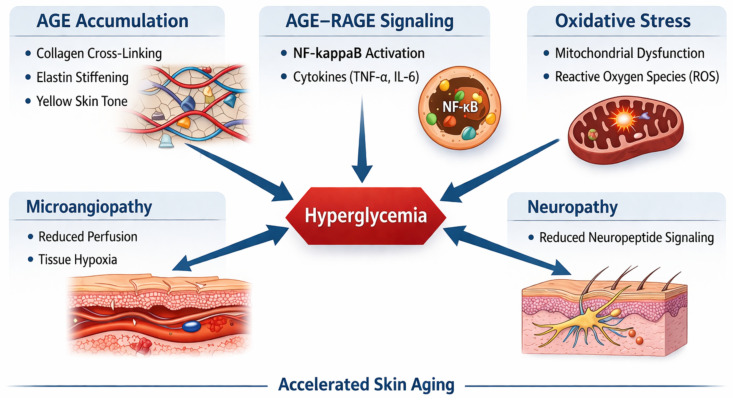
Hyperglycemia-driven mechanisms of accelerated skin aging in diabetes. Chronic hyperglycemia promotes advanced glycation end product (AGE) formation and collagen cross-linking, increasing dermal stiffness and impairing matrix turnover. AGE–RAGE signaling activates NF-κB-mediated inflammation and oxidative stress, while diabetic microangiopathy and neuropathy reduce tissue perfusion and neurotrophic support. These interconnected pathways contribute to accelerated skin aging. Abbreviations: AGE, advanced glycation end product; RAGE, receptor for advanced glycation end products; NF-κB, nuclear factor kappa B. Created in BioRender. Mittal, R. (2026) https://BioRender.com/puijm1y.

Diabetes exerts multisystem effects on the skin that go well beyond the wound-healing complications that dominate clinical literature. As many as 70% of patients with diabetes develop cutaneous manifestations during their disease course [3,4,5]. These include not only medically significant conditions such as diabetic foot ulcers and recurrent infections, but also cosmetically distressing changes such as premature wrinkling, loss of skin elasticity, xerosis, sallow or yellow skin discoloration, dyschromia, alopecia, skin tags, acanthosis nigricans, and a general appearance of accelerated aging [6,7,8] (Figure 1). These aesthetic concerns carry meaningful psychological consequences, affecting self-esteem, social functioning, body image, and health-related quality of life [9,10,11,12].

Despite this, cosmetic dermatology literature has largely been developed for metabolically healthy populations. Studies of aesthetic procedures including neuromodulators, dermal fillers, chemical peels, laser therapies, and microneedling routinely exclude patients with uncontrolled diabetes, and few have specifically evaluated outcomes in well controlled diabetic cohorts [13,14,15]. Meanwhile, regenerative medicine has generated considerable preclinical and early clinical evidence for platelet-derived and mesenchymal stromal cell (MSC)–based therapies in tissue repair, but the application of these modalities to cosmetic rather than wound-healing indications in diabetes remains largely unexplored.

This narrative review addresses these gaps by integrating cosmetic dermatologic practice within the biological framework of diabetes-associated skin aging and repair dysfunction. Prior literature has generally examined diabetic skin disease, aesthetic interventions, and regenerative therapies as distinct areas of investigation, without systematically linking disease-specific mechanisms to procedural outcomes. Although emerging work has considered the safety of aesthetic procedures in patients with diabetes, these analyses have not incorporated a mechanistic evaluation of how diabetes-related alterations in tissue biology influence treatment response, complication risk, and therapeutic optimization.

Accordingly, the present narrative review provides a unified synthesis that connects key molecular drivers of diabetic skin aging, including advanced glycation end product accumulation, chronic inflammation, oxidative stress, and microvascular dysfunction, with clinically relevant changes in structural integrity, pigmentation, and regenerative capacity. It further evaluates both conventional aesthetic procedures and emerging regenerative modalities within this context, with emphasis on how metabolic dysregulation may modify efficacy and safety profiles. Building on this integration, a structured, clinically oriented framework is proposed that incorporates metabolic assessment, dermatologic evaluation, risk stratification, and procedure-specific modifications to guide aesthetic care in patients with diabetes.

## 2. Methods

This review was conducted as a structured narrative synthesis of the literature to examine the intersection of diabetes mellitus, skin aging, cosmetic dermatologic procedures, and regenerative therapies. A comprehensive literature search was performed using the electronic databases PubMed/MEDLINE, Embase, and Scopus to identify relevant publications.

Search strategies incorporated controlled vocabulary and keyword combinations related to diabetes and aesthetic dermatology, including “diabetes mellitus,” “skin aging,” “cutaneous aging,” “cosmetic dermatology,” “aesthetic procedures,” “dermal fillers,” “neuromodulators,” “chemical peels,” “laser therapy,” “microneedling,” “platelet-rich plasma,” “platelet lysate,” “exosomes,” and “mesenchymal stromal cells.” Boolean operators (“AND,” “OR”) were used to combine terms and refine results. Reference lists of selected articles were also screened to identify additional relevant studies.

Studies were included based on relevance to one or more of the following domains: (i) pathophysiology of diabetes-associated skin aging, (ii) safety and efficacy of aesthetic dermatologic procedures in patients with diabetes, and (iii) regenerative therapies with potential cosmetic applications. Both preclinical and clinical studies were considered to provide a comprehensive translational perspective. Priority was given to peer-reviewed original research, systematic reviews, and high-quality narrative reviews.

Exclusion criteria included studies not involving cutaneous or aesthetic outcomes, and reports lacking sufficient methodological detail. Given the limited availability of diabetes-specific data in aesthetic dermatology, studies conducted in non-diabetic populations were included where necessary to contextualize procedural mechanisms and expected outcomes.

The literature search primarily focused on studies published within the past 20 years, with earlier seminal publications included where relevant to foundational biological mechanisms. Due to heterogeneity in study design, outcome measures, and reporting standards, a quantitative meta-analysis was not performed. Instead, findings were synthesized qualitatively, with emphasis on mechanistic integration and clinical applicability.

## 3. Skin Aging: Chronological, Photoaging, and Diabetes-Accelerated Pathways

**Figure 2:**
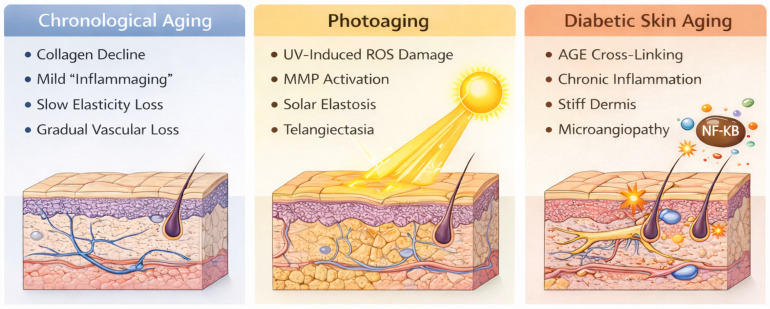
Mechanistic pathways of chronological, photoinduced, and diabetes-associated skin aging. Schematic comparison of the principal biological mechanisms underlying intrinsic aging, ultraviolet (UV)-induced photoaging, and diabetes-associated skin aging. Chronological aging involves gradual collagen decline, mild inflammaging, loss of dermal elasticity, and vascular reduction. Photoaging is driven by UV-induced reactive oxygen species (ROS), matrix metalloproteinase (MMP) activation, solar elastosis, and telangiectasia. Diabetes-associated skin aging is characterized by accelerated advanced glycation end product (AGE) accumulation, NF-κB-mediated chronic inflammation, dermal collagen cross-linking and stiffening, and microangiopathy. Abbreviations: UV, ultraviolet; ROS, reactive oxygen species; MMP, matrix metalloproteinase; AGE, advanced glycation end product; NF-κB, nuclear factor kappa B. Created in BioRender. Mittal, R. (2026) https://BioRender.com/c5hlu32.

Understanding the distinctions between chronological aging, photoaging, and diabetes-accelerated skin aging is essential for tailoring aesthetic strategies (Table 1). Table 1 summarizes the key mechanistic and clinical differences among these processes, highlighting features of direct relevance to clinical practice. In particular, diabetic skin is characterized by accelerated advanced glycation end product accumulation, chronic inflammation, and microvascular dysfunction, which collectively contribute to impaired healing and increased procedural risk. These distinctions highlights the need for modified aesthetic approaches in this population. Although these processes share overlapping molecular pathways, their relative contributions, kinetics, and clinical manifestations differ in ways that are directly relevant to treatment selection and expected outcomes (Figure 2).

### 3.1. Chronological (Intrinsic) Aging

Chronological aging is a genetically programmed, time-dependent process affecting all skin structures. Key features include progressive epidermal thinning (approximately 6.4% per decade after age 30), flattening of the dermal–epidermal junction with loss of rete ridges, decreased melanocyte density (approximately 8–10% per decade), reduced fibroblast number and synthetic activity, and gradual decline in dermal collagen (approximately 1% per year in adulthood) [16,17]. Elastin fibers undergo slow fragmentation, and glycosaminoglycan content diminishes, reducing dermal hydration and turgor [20,31,32]. Cutaneous microvasculature decreases in density by approximately 30–40% between the third and eighth decades, and sensory nerve fiber density declines progressively [16,27,33]. These changes collectively produce fine wrinkling, mild laxity, dryness, and pallor, but the process is gradual, and the dermis retains substantial capacity for repair and remodeling well into advanced age.

### 3.2. Photoaging (Extrinsic Aging)

Ultraviolet radiation, particularly UVA induces cumulative damage that superimposes upon and accelerates chronological aging [18]. Photoaging is characterized by thickened, leathery skin texture (solar elastosis), coarse wrinkling, mottled hyperpigmentation and hypopigmentation, telangiectasia, and actinic keratoses [25,34,35]. At the molecular level, UV exposure generates reactive oxygen species (ROS), activates matrix metalloproteinases (MMPs) that degrade collagen and elastin, and induces chronic subclinical inflammation [21,23,36]. Importantly, UV exposure also accelerates the formation of advanced glycation end products (AGEs) in dermal proteins, creating a mechanistic link between photoaging and glycation-driven aging.

### 3.3. Diabetes-Accelerated Skin Aging

Diabetic skin aging encompasses all features of chronological and photoaging but adds several distinct, disease-specific processes that accelerate and amplify structural and functional decline. The key distinguishing mechanisms are discussed below.

#### 3.3.1. Advanced Glycation End Products (AGEs)

AGEs are formed through the non-enzymatic Maillard reaction between reducing sugars and free amino groups on proteins, lipids, and nucleic acids [37,38]. In healthy individuals, AGEs accumulate slowly with age; skin collagen AGE levels (carboxymethyllysine, pentosidine, glucosepane) increase linearly over the lifespan [39,40,41,42]. In diabetes, chronic hyperglycemia dramatically accelerates this process, producing AGE levels in skin collagen that are severalfold higher than age-matched non-diabetic controls [38,43]. AGEs cross-link collagen and elastin fibers, producing stiffness, loss of elasticity, resistance to enzymatic turnover, and impaired matrix remodeling [44]. Clinically, this manifests as premature wrinkling, reduced skin pliability, and a characteristic yellowed or sallow complexion due to the inherent brown pigmentation of AGE-modified proteins [22]. AGEs also activate the receptor for AGEs (RAGE) on fibroblasts, keratinocytes, and endothelial cells, triggering NF-κB signaling, pro-inflammatory cytokine release, ROS generation, and MMP expression—creating a self-amplifying cycle of matrix destruction and chronic inflammation [23,24].

#### 3.3.2. Chronic Low-Grade Inflammation

Diabetes establishes a persistent inflammatory microenvironment in the skin characterized by elevated TNF-α, IL-6, IL-1β, and C-reactive protein [45,46,47]. Macrophages in diabetic skin are skewed toward a pro-inflammatory M1 phenotype and fail to transition to the M2 reparative state [48,49,50]. This chronic inflammation accelerates fibroblast senescence, suppresses collagen synthesis, upregulates MMPs, and impairs the resolution of tissue damage following aesthetic procedures [51,52,53].

#### 3.3.3. Oxidative Stress and Mitochondrial Dysfunction

Hyperglycemia drives excessive ROS production through multiple pathways: the polyol pathway, protein kinase C activation, hexosamine pathway, and AGE–RAGE signaling [54,55]. Diabetic skin fibroblasts and keratinocytes exhibit elevated oxidative stress markers, mitochondrial dysfunction, and impaired antioxidant defenses [56,57]. This accelerates telomere shortening, induces premature cellular senescence, and reduces the regenerative capacity of skin stem and progenitor cells [58,59].

#### 3.3.4. Microangiopathy and Tissue Hypoxia

Diabetic microangiopathy is characterized by capillary basement membrane thickening, reduced capillary density, endothelial dysfunction, and impaired vasoreactivity diminishes cutaneous perfusion and oxygen delivery [31,32]. This has direct implications for aesthetic procedures that depend on adequate blood supply for healing (laser resurfacing, chemical peels, surgical procedures) and for the delivery of growth factors and regenerative signals to the dermis.

#### 3.3.5. Peripheral Neuropathy

Diabetic peripheral neuropathy reduces sensory, motor, and autonomic nerve fiber density in the skin. Neuropeptides normally released by cutaneous nerves including substance P, calcitonin gene related peptide (CGRP), and neuropeptide Y modulate keratinocyte migration, angiogenesis, and immune responses [33]. Their reduction in diabetic skin impairs these neurotrophic signaling pathways and reduces the skin’s capacity to respond to controlled injury the principle underlying many aesthetic procedures [34].

#### 3.3.6. Barrier Dysfunction

Diabetic skin exhibits impaired barrier function with decreased stratum corneum hydration, increased transepidermal water loss (TEWL), reduced ceramide and cholesterol content in the lipid lamellae, and diminished sebaceous gland activity [35,36]. Clinically, this produces the characteristic xerosis of diabetic skin, but it also increases susceptibility to irritant reactions from topical agents and may compromise the recovery of the epidermal barrier following ablative procedures.

### 3.4. Skin Microbiome in Skin Aging and Diabetes-Associated Cutaneous Dysfunction

#### 3.4.1. Role of the Skin Microbiome in Cutaneous Homeostasis and Aging

The skin microbiome represents a dynamic and functionally active component of the cutaneous microenvironment that contributes to epidermal barrier integrity, immune regulation, and maintenance of tissue homeostasis [60,61]. Commensal microorganisms regulate keratinocyte differentiation, lipid metabolism, and antimicrobial peptide production, thereby supporting barrier function and modulating local inflammatory responses. Disruption of this equilibrium has been associated with increased transepidermal water loss, heightened inflammatory signaling, and impaired regenerative capacity.

Emerging evidence indicates that the microbiome participates directly in biological processes underlying skin aging. Microbial-derived metabolites and host–microbe signaling interactions influence oxidative stress pathways, inflammatory cascades, and extracellular matrix turnover, thereby contributing to collagen degradation and structural changes associated with aging [60,61].

#### 3.4.2. Age-Associated Microbiome Alterations

Age-related changes in the skin microbiome are characterized by shifts in microbial diversity, taxonomic composition, and ecological stability. Human studies have demonstrated that individuals with premature skin aging exhibit distinct microbial signatures and reduced microbial network robustness compared with age-matched controls. In one study, enrichment of Acinetobacter species and disruption of microbial interaction networks were associated with features of premature aging, while topical retinoid therapy was accompanied by improvements in skin physiology and partial normalization of microbial composition [62].

Integrative analyses combining microbiome profiling with biophysical skin parameters have further demonstrated correlations between microbial composition and measures such as hydration, elasticity, and sebum production, supporting a functional relationship between microbial ecology and structural skin properties [63]. Additional studies indicate that microbiome aging signatures vary by anatomical site and environmental exposure, reflecting the influence of local skin physiology and external factors [64].

#### 3.4.3. Microbiome-Derived Mechanisms Relevant to Skin Aging

Mechanistic studies have identified specific microbial taxa and metabolites that influence pathways central to cutaneous aging. Pantothenate-producing Rothia species have demonstrated anti-inflammatory and photoaging-protective effects, supporting a role for microbiome-derived vitamins in maintaining dermal homeostasis [65]. Microbiota-derived porphyrins have been implicated in the regulation of melanogenesis and age-associated pigmentary changes, suggesting a link between microbial metabolism and dyschromia [66].

Multi-omics investigations have further demonstrated coordinated interactions between host barrier function and microbial metabolic activity. Retinoid-induced barrier adaptation has been associated with restructuring of host–microbiome metabolic networks, indicating that therapeutic modulation of the skin may involve parallel microbiome remodeling [67].

Interventional studies further support functional relevance. Topical probiotics and bacterial lysates have been associated with improvements in hydration, elasticity, and wrinkle appearance, as well as reductions in inflammatory signaling [68,69]. Microbiome-derived yeast extracts enriched in antioxidant systems have demonstrated enhancement of collagen content and attenuation of oxidative stress [70], while fermentation-derived topical formulations have been shown to improve barrier lipid composition and modulate microbial communities [71].

#### 3.4.4. Microbiome Dysregulation in Diabetes

In diabetes mellitus, alterations in the skin microbiome appear to be more pronounced and may interact with established mechanisms of cutaneous dysfunction. Recent evidence demonstrates associations between microbial composition and inflammatory cytokine profiles in individuals with type 2 diabetes, suggesting that dysbiosis may contribute to the chronic inflammatory state characteristic of the disease [72].

These microbiome alterations occur within a metabolically altered cutaneous environment characterized by hyperglycemia, advanced glycation end product accumulation, oxidative stress, microangiopathy, and impaired barrier function [60,61,72]. This environment may favor colonization by opportunistic organisms, reduce microbial diversity, and amplify inflammatory signaling pathways [61]. In turn, microbiome dysregulation may impair barrier recovery and regenerative responses following controlled injury, with direct implications for aesthetic procedures that depend on predictable wound healing and collagen remodeling [62].

Taken together, incorporation of the skin microbiome expands the mechanistic framework of diabetes-associated skin aging by introducing a bidirectional interface between metabolic dysfunction and cutaneous homeostasis. However, clinical evidence evaluating microbiome-targeted interventions specifically in aesthetic dermatology for patients with diabetes remains limited.

## 4. Diabetes-Specific Aesthetic Concerns

Beyond the accelerated aging features described above, patients with diabetes present with a constellation of cosmetically significant cutaneous conditions that are either unique to or disproportionately prevalent in this population. Understanding these conditions is essential for comprehensive aesthetic assessment and treatment planning.

### 4.1. Xerosis and Textural Changes

Xerosis is the most common dermatologic finding in diabetes, affecting 40–70% of patients [28,29]. Diabetic xerosis is more severe than age-related dry skin, characterized by markedly reduced stratum corneum hydration, decreased ceramide synthesis, and impaired lamellar body formation. Clinically, the skin appears rough, scaly, and dull, and patients frequently report pruritus. These textural changes affect cosmetic outcomes by altering how the skin responds to topical agents, peels, and energy-based devices, and by reducing the aesthetic baseline against which procedural improvements are measured.

### 4.2. Dyschromia and Pigmentary Changes

Patients with diabetes exhibit several forms of dyschromia relevant to cosmetic practice. AGE accumulation produces a characteristic sallow or yellow skin tone distinct from age-related pallor [73]. Acanthosis nigricans hyperpigmented, velvety plaques in intertriginous areas is strongly associated with insulin resistance and affects up to 74% of obese patients with type 2 diabetes [74]. Diabetic dermopathy produces light brown, atrophic macules on the shins, often mistaken for age spots [75]. Additionally, AGE-mediated activation of RAGE in melanocytes stimulates melanogenesis through ERK (extracellular signal-regulated kinase) and CREB (cAMP response element-binding protein) signaling pathways, contributing to hyperpigmentation [76]. Vitiligo is more common in type 1 diabetes through shared autoimmune mechanisms [77]. These diverse pigmentary disturbances require targeted assessment and may influence the choice and settings of pigment-directed laser and light-based therapies. Notably, patients with skin of color and concurrent type 2 diabetes may experience disproportionately severe glycation-mediated hyperpigmentation due to higher baseline melanin and fibroblast activity, warranting particular attention in this population [78].

### 4.3. Premature Wrinkling and Loss of Elasticity

Studies using biophysical measurement tools (cutometry, profilometry, reflectance confocal microscopy) have demonstrated that patients with diabetes exhibit significantly deeper and more voluminous wrinkles, reduced skin elasticity, and more deformed collagen fibers compared with age-matched controls [6,26]. Reflectance confocal microscopy reveals polycyclic dermal papillae and amorphous collagen architecture in diabetic skin features not seen in healthy controls of similar age [6]. These structural changes have direct implications for the efficacy of procedures that rely on collagen stimulation (microneedling, fractional laser, radiofrequency) and volume restoration (dermal fillers), as the underlying dermal matrix may respond differently to controlled injury in diabetic skin.

### 4.4. Hair Loss

Hair thinning and alopecia are underrecognized complications of diabetes, affecting both men and women. Mechanisms include microangiopathy of the scalp vasculature, oxidative stress-induced damage to hair follicle stem cells, hormonal dysregulation (hyperandrogenism in type 2 diabetes, thyroid comorbidity in type 1 diabetes), and insulin resistance effects on the hair growth cycle [79,80]. Telogen effluvium associated with poor glycemic control is also well documented. These hair loss patterns create demand for regenerative hair restoration therapies, but the efficacy of platelet-rich plasma (PRP) and other regenerative approaches may be modified by the underlying metabolic abnormalities.

### 4.5. Skin Tags, Rubeosis Faciei, and Other Cosmetic Concerns

Acrochordons (skin tags) are significantly more prevalent in patients with diabetes and insulin resistance, occurring in friction-prone areas such as the neck, axillae, and groin [81]. While medically benign, they are among the most common cosmetic complaints in this population. Rubeosis faciei persistent facial erythema from microangiopathy can mimic rosacea and complicate the aesthetic assessment [82]. Eruptive xanthomas, carotenodermia (yellowish palms and soles), and granuloma annulare further expand the cosmetically relevant dermatologic landscape in diabetes [75,83].

## 5. Conventional Aesthetic Procedures in Patients with Diabetes: Safety and Efficacy Considerations

Diabetes modifies the risk–benefit profile of virtually every aesthetic procedure through its effects on healing, infection susceptibility, vascular supply, collagen metabolism, and pigmentation (Table 2). Table 2 provides an overview of the relative risk profiles and key considerations for commonly performed aesthetic procedures in patients with diabetes mellitus. The stratification highlights how diabetes-related alterations in healing, immune function, and vascular integrity influence both safety and efficacy, supporting the need for individualized treatment planning and careful patient selection. Fritz et al. (2025) provided the first comprehensive overview of diabetes’s impact on aesthetic procedures, emphasizing that delayed healing, altered collagen metabolism, vascular complications, and immune compromise collectively influence both safety and long-term outcomes [84]. The following section examines procedure-specific considerations.

### 5.1. Neuromodulators (Botulinum Toxin)

Botulinum toxin injections are generally considered among the safest aesthetic procedures for patients with diabetes. The mechanism neuromuscular blockade does not rely on dermal healing or collagen remodeling. However, diabetic peripheral neuropathy may theoretically alter neuromuscular junction sensitivity, and microangiopathy could increase bruising risk at injection sites [84]. No large studies have specifically evaluated botulinum toxin outcomes in diabetic versus non-diabetic populations, representing an evidence gap. Clinical experience suggests that outcomes are generally comparable, but practitioners should be attentive to altered bruising patterns and potentially modified duration of effect.

### 5.2. Dermal Fillers

Hyaluronic acid (HA) fillers are widely used for volume restoration, but diabetes raises several concerns. Altered collagen metabolism may affect the longevity of biostimulatory fillers (e.g., poly-L-lactic acid, calcium hydroxylapatite) that depend on neocollagenesis for sustained results [84,85]. AGE-modified collagen may respond differently to the mechanical and biological stimuli generated by filler placement. Immunocompromise increases the risk of biofilm formation and late-onset inflammatory reactions. Studies from the CosmetAssure database of over 129,000 aesthetic surgical patients have identified diabetes as an independent risk factor for major complications following cosmetic procedures, though filler-specific data are limited [86]. Pre-procedural glycemic optimization and strict aseptic techniques are essential.

### 5.3. Chemical Peels

Chemical peels create controlled epidermal and dermal injury to stimulate regeneration. In diabetic skin, delayed re-epithelialization, impaired barrier recovery, and increased infection risk are significant concerns, particularly with medium-depth and deep peels [84,87]. Superficial peels (glycolic acid, salicylic acid) are generally safer and may additionally provide benefit through their keratolytic and humectant properties in xerotic diabetic skin. Medium-depth peels (trichloroacetic acid 35%) require careful patient selection and post-procedural monitoring. Deep peels (phenol) carry substantially elevated risk in diabetic patients and are generally contraindicated in the setting of poor glycemic control [87].

### 5.4. Laser and Energy-Based Devices

Laser therapies span a wide spectrum of risk in diabetic patients. Non-ablative fractional lasers, which leave the stratum corneum intact and rely on dermal collagen stimulation, carry a more favorable risk profile than ablative devices [88]. Ablative fractional and fully ablative lasers (CO_2_, erbium:YAG) create open wounds and carry heightened risk of delayed healing, infection, and scarring in the setting of diabetic microangiopathy and immune compromise [84]. Vascular lasers (pulsed dye, potassium titanyl phosphate (KTP) laser) are generally safe but may show altered efficacy when targeting the dysfunctional microvasculature of diabetic skin. Intense pulsed light (IPL) for pigmentary concerns may require modified parameters given the AGE-mediated pigmentary changes unique to diabetes. Radiofrequency and ultrasound devices for skin tightening rely on controlled thermal injury to stimulate neocollagenesis; their efficacy in the context of AGE-stiffened, poorly vascularized diabetic dermis has not been specifically studied.

### 5.5. Microneedling

Microneedling creates controlled dermal micro-injuries to stimulate collagen induction through the wound-healing cascade. In healthy populations, microneedling is well-established for skin rejuvenation, acne scarring, and as a transdermal delivery system [89]. In diabetic patients, the impaired wound-healing response raises questions about whether the collagen induction stimulus is adequately translated into neocollagenesis. Conversely, microneedling combined with regenerative agents (PRP, growth factor serums) may partially compensate for the endogenous signaling deficits of diabetic skin. Studies of microneedling for hair loss consistently exclude patients with uncontrolled diabetes, but do not specifically report outcomes in well-controlled diabetic subgroups [90,91]. Needle depth, treatment intervals, and infection prophylaxis protocols may need modification for diabetic patients.

## 6. Regenerative Therapies for Cosmetic Applications in Diabetes

**Figure 3:**
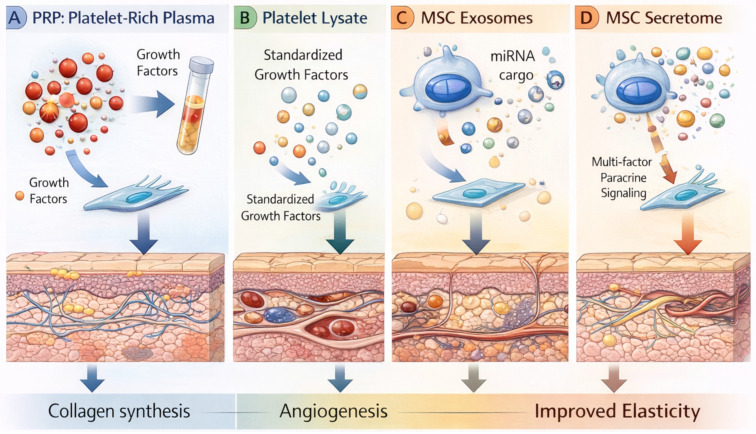
Regenerative biologic therapies in skin repair and aesthetic applications. (**A**) Platelet-rich plasma (PRP) delivers autologous platelet-derived growth factors that stimulate fibroblast activation and collagen synthesis. (**B**) Platelet lysate provides a standardized source of platelet-derived growth factors that support tissue repair and angiogenesis. (**C**) Mesenchymal stem cell (MSC)–derived exosomes transfer bioactive cargo, including microRNAs and proteins, that modulate cellular signaling and promote regeneration. (**D**) The MSC secretome contains a complex mixture of cytokines, growth factors, and extracellular vesicles that act through paracrine signaling to enhance dermal remodeling and vascularization. Collectively, these regenerative approaches promote collagen production, angiogenesis, and improved skin elasticity. Abbreviations: PRP, platelet-rich plasma; MSC, mesenchymal stromal cell. Created in BioRender. Mittal, R. (2026) https://BioRender.com/5140tou.

Regenerative therapies offer a biologically rational approach to cosmetic care in diabetic patients because they target the underlying molecular deficits such as growth factor resistance, impaired angiogenesis, chronic inflammation, and stem cell exhaustion rather than relying solely on the skin’s endogenous healing capacity, which is compromised in diabetes (Figure 3). This section evaluates each major regenerative modality with respect to its potential cosmetic applications, diabetes-specific considerations, and current evidence base (Table 3). Table 3 summarizes the regenerative therapy modalities and their potential applications in aesthetic dermatology for patients with diabetes. While these approaches offer biologically targeted strategies to address impaired regeneration, the table also emphasizes the limited clinical evidence and the need for cautious interpretation, particularly when extrapolating from non-diabetic populations.

A critical limitation of the current evidence base is that the majority of data supporting regenerative therapies in aesthetic dermatology are derived from studies conducted in metabolically healthy populations, with limited direct evaluation in patients with diabetes mellitus. Consequently, extrapolation of efficacy and safety outcomes to diabetic skin requires careful interpretation in light of disease-specific alterations in inflammation, angiogenesis, and tissue repair.

While regenerative therapies offer a biologically rational approach to addressing impaired cutaneous repair in diabetes, the level of clinical evidence varies substantially across modalities. Among these, platelet-rich plasma (PRP) represents the most clinically utilized approach in aesthetic dermatology, although existing studies remain heterogeneous and highly protocol-dependent, with limited data in diabetic populations. In contrast, platelet lysate, mesenchymal stromal cell (MSC)–derived exosomes, and MSC secretomes should be considered experimental or early translational modalities for cosmetic applications in diabetes. Current evidence for these approaches is derived predominantly from in vitro, animal, or wound-healing studies rather than controlled clinical trials in aesthetic settings. Accordingly, their potential benefits in cosmetic dermatology remain theoretical and require cautious interpretation.

### 6.1. Platelet-Rich Plasma (PRP)

PRP has become the most widely used regenerative modality in aesthetic dermatology, with applications in facial rejuvenation, hair restoration, scar remodeling, and as an adjunct to microneedling and laser therapy [101,102]. PRP delivers a concentrated bolus of autologous growth factors platelet-derived growth factor (PDGF), transforming growth factor beta (TGF-β), vascular endothelial growth factor (VEGF), epidermal growth factor (EGF), and others that stimulate fibroblast activity, collagen synthesis, angiogenesis, and stem cell activation [103].

In diabetic patients, several considerations modify the expected outcomes. Platelet function is altered in diabetes: platelets exhibit hyperreactivity with paradoxically impaired degranulation of specific growth factor pools, altered activation kinetics, and modified surface receptor expression [104]. These changes may reduce the regenerative potency of autologous PRP. Systematic reviews in aesthetic applications report mean increases of 18–27.7 hairs/cm^2^ for PRP in androgenetic alopecia, but these studies largely exclude diabetic patients or do not report diabetic subgroup analyses [101,102].

In diabetic skin, the biological activity of PRP is modulated by hyperglycemia-associated alterations in platelet function and growth factor signaling, with evidence demonstrating impaired production, release kinetics, and cellular responsiveness to key mediators of tissue repair [105,106]. Platelets in diabetes exhibit increased basal activation accompanied by dysregulated degranulation, resulting in altered release profiles of platelet-derived growth factor, transforming growth factor-β, and vascular endothelial growth factor, which are essential for angiogenesis and extracellular matrix remodeling [106].

Chronic hyperglycemia and advanced glycation end product accumulation further disrupt receptor-mediated signaling pathways, including PI3K/Akt and MAPK cascades, thereby attenuating fibroblast proliferation, impairing collagen synthesis, and reducing cellular responsiveness to exogenous growth factors delivered through PRP [107,108]. These signaling abnormalities are compounded by oxidative stress and mitochondrial dysfunction, which impair endothelial cell migration and angiogenic capacity, limiting neovascularization and regenerative efficiency following PRP administration [109,110].

Within this metabolically altered microenvironment, the therapeutic effects of PRP are constrained not only by variability in growth factor delivery but also by intrinsic defects in cellular signaling, metabolic regulation, and tissue responsiveness that characterize diabetic skin [30,111].

Limitations: High variability in PRP preparation protocols limits cross-study comparison. Diabetes-specific platelet dysfunction may reduce autologous PRP potency, but this has not been quantified in aesthetic applications. No randomized controlled trials have evaluated PRP for cosmetic indications specifically in diabetic cohorts. Notably, most clinical evidence supporting PRP in aesthetic applications is derived from non-diabetic populations, and the extent to which these findings translate to diabetic skin, characterized by altered platelet function and impaired regenerative signaling, remains unclear.

### 6.2. Platelet Lysate

Platelet lysate produced by freeze thaw lysis of platelets offers potential advantages over PRP in the diabetic cosmetic context. The cell-free product provides more complete and reproducible growth factor release, can be manufactured from pooled allogeneic donors (bypassing patient-specific platelet dysfunction), and is amenable to standardization, lyophilization, and quality-controlled manufacturing [112,113]. Platelet lysate comprises a concentrated repertoire of platelet-derived growth factors, cytokines, and extracellular vesicle-associated signaling mediators with enhanced bioavailability compared with platelet-rich plasma [114,115]. This facilitates more efficient activation of cellular repair pathways in metabolically impaired tissues. In diabetic skin, chronic hyperglycemia induces advanced glycation end-product accumulation and oxidative stress, which disrupt growth factor receptor signaling and attenuate downstream pathways regulating fibroblast proliferation, migration, and extracellular matrix synthesis [116,117,118]. Exogenous administration of platelet lysate can partially restore impaired signaling by enhancing ligand–receptor interactions and reactivating intracellular pathways governing cellular proliferation and matrix production in hyperglycemic microenvironments [119,120,121,122]. Platelet lysate additionally modulates mesenchymal stem cell function by reducing senescence-associated changes and enhancing paracrine secretion of pro-angiogenic and matrix-regulatory factors, thereby supporting tissue remodeling in diabetic conditions [123,124,125,126]. In vitro, platelet lysate has demonstrated superior stimulation of fibroblast proliferation and extracellular matrix synthesis compared with PRP [127]. For cosmetic applications, platelet lysate could serve as a topical or injectable growth factor supplement to enhance the outcomes of microneedling, fractional laser, and other collagen-induction procedures.

Limitations: Clinical evidence for platelet lysate in cosmetic dermatology is sparse and largely derived from wound-healing studies. Allogeneic products carry theoretical immunogenicity concerns that require characterization. No studies have evaluated topical or injectable platelet lysate for facial rejuvenation or hair restoration in diabetic patients specifically. Furthermore, available data are predominantly derived from non-diabetic experimental or wound-healing models, and their relevance to cosmetic outcomes in diabetic populations has not been directly established.

### 6.3. MSC-Derived Exosomes

Exosomes (30–150 nm extracellular vesicles) derived from mesenchymal stromal cells carry a cargo of microRNAs, proteins, and lipids that modulate gene expression and signaling in recipient cells [128]. Preclinical studies have demonstrated that MSC-derived exosomes stimulate collagen synthesis, enhance angiogenesis, promote keratinocyte proliferation, modulate inflammation, and protect against oxidative stress [98,129]. In the cosmetic context, exosomes are being explored as topical adjuncts to microneedling and laser therapy, as injectables for skin rejuvenation, and as components of cosmeceutical formulations [130].

The engineerability of exosomes including cargo enrichment with specific anti-aging microRNAs, anti-glycation peptides, or antioxidant molecules is particularly attractive for diabetic cosmetic applications where targeting advanced glycation end products (AGEs) mediated damage and oxidative stress is desirable [131]. Exosome-loaded hydrogels and biomaterial carriers could provide sustained delivery of regenerative signals to the dermis.

MSC-derived exosomes exert regenerative effects through the transfer of microRNAs, proteins, and lipids that regulate angiogenesis, inflammation, and extracellular matrix remodeling [132,133,134]. However, the diabetic microenvironment significantly alters exosome uptake and downstream signaling in recipient skin cells. Hyperglycemia impairs exosome-mediated signaling by disrupting key pathways such as Wnt/β-catenin, PI3K/Akt, and Notch, leading to reduced keratinocyte migration, diminished angiogenesis, and delayed re-epithelialization in diabetic skin models [133,135,136,137]. Furthermore, diabetic conditions alter the cargo composition of MSC-derived exosomes, including reduced levels of pro-angiogenic microRNAs (e.g., miR-126, miR-21) and increased pro-inflammatory mediators, thereby limiting their regenerative efficacy in cutaneous applications [135,138].

Importantly, despite these diabetes-associated impairments, MSC-derived exosomes have demonstrated beneficial effects in diabetic wound models by enhancing angiogenesis and promoting keratinocyte migration and re-epithelialization [134,139]. They also facilitate macrophage polarization toward reparative phenotypes and improve collagen deposition and overall wound closure [134,140]. These findings support cautious translational interest in regenerative dermatologic applications [141,142,143].

Limitations: The exosome field faces significant challenges in standardization, isolation methodology, quantification, and quality control [144]. MISEV2018 guidelines have improved reporting but have not fully resolved reproducibility concerns. The regulatory landscape for cosmetic exosome products is uncertain and evolving. Most evidence is preclinical; clinical data for cosmetic applications are extremely limited, and no studies have evaluated exosomes for aesthetic indications in diabetic patients. The commercial marketing of exosome-based cosmetic products has outpaced the clinical evidence, and practitioners should exercise caution regarding unregulated products [130]. Importantly, current evidence is primarily based on preclinical studies or non-diabetic cohorts, limiting the ability to extrapolate efficacy and safety to diabetic patients.

### 6.4. MSC-Derived Secretomes

The MSC-derived secretome provides a broader regenerative effect than single-agent therapies. It contains cytokines, growth factors, and extracellular vesicles that act together to restore tissue homeostasis in diabetic skin.

Defined secretomes encompass the full paracrine output of MSCs under controlled culture conditions, including soluble proteins, extracellular vesicles, lipid mediators, and metabolites [145,146]. The broader signaling repertoire compared with isolated exosomes may be advantageous in diabetic skin, where multiple pathways are simultaneously dysregulated. Preclinical evidence supports secretome-mediated improvements in collagen synthesis, anti-inflammatory macrophage polarization, antioxidant protection, and re-epithelialization [146,147].

Xeno-free, umbilical tissue–derived MSC secretomes have been proposed as reproducible, off-the-shelf regenerative products with favorable immunologic profiles [148]. For cosmetic applications, secretomes could be delivered topically (with microneedling-assisted penetration), by intradermal injection, or incorporated into cosmeceutical formulations.

The MSC secretome comprises a complex mixture of soluble cytokines, chemokines, growth factors, and extracellular vesicles that act through paracrine signaling to regulate inflammation, angiogenesis, and matrix remodeling [149,150,151]. However, diabetic metabolic conditions significantly disrupt these signaling networks in cutaneous tissues [152,153]. Hyperglycemia induces alterations in MSC secretory profiles, including reduced secretion of VEGF, hepatocyte growth factor, and insulin-like growth factor-1, alongside increased pro-inflammatory cytokines, thereby impairing angiogenic and regenerative responses in diabetic skin [154,155,156]. In addition, advanced glycation end products and oxidative stress impair MSC paracrine signaling by modifying protein structure and receptor interactions, leading to reduced activation of downstream repair pathways and diminished dermal regeneration capacity [157,158,159].

Limitations: All limitations described for exosomes apply with additional concerns. Secretome composition is highly sensitive to culture conditions, and batch-to-batch variability remains a major translational challenge [160,161]. The immunogenicity of allogeneic secretome proteins with repeated application has not been characterized. Clinical evidence is predominantly preclinical and early-phase. No validated potency assays exist for cosmetic secretome applications. Claims of superiority over other regenerative modalities are not supported by comparative clinical data [146]. In addition, the absence of studies specifically evaluating secretome-based therapies in diabetic populations represents a significant gap, particularly given the altered inflammatory and regenerative milieu of diabetic skin. Furthermore, MSC secretome remains an emerging experimental modality, with limited clinical validation and significant variability in composition and standardization.

### 6.5. Critical Appraisal of Evidence and Limitations

Despite increasing interest in regenerative therapies for aesthetic dermatology, the overall strength of evidence remains limited and heterogeneous, particularly in the context of diabetes mellitus. Much of the available literature is derived from preclinical investigations, small observational studies, or clinical trials conducted in metabolically healthy populations. As a result, the applicability of these findings to patients with diabetes remains uncertain, given the well-established alterations in inflammation, angiogenesis, cellular signaling, and tissue repair associated with the disease.

Significant methodological variability exists across studies evaluating regenerative interventions. For PRP, differences in preparation protocols, platelet concentration, activation techniques, and delivery methods contribute to inconsistent outcomes and limit reproducibility. Reported clinical effects range from modest improvements in skin texture and elasticity to more pronounced regenerative responses, suggesting that both technical factors and patient-specific variables influence therapeutic efficacy.

The evidence base for platelet lysate is comparatively less developed, with most data derived from in vitro models and wound-healing applications rather than aesthetic indications. Although these studies demonstrate enhanced cellular proliferation and extracellular matrix synthesis, clinical translation to cosmetic outcomes remains insufficiently characterized. Similarly, MSC-derived exosomes and secretomes have shown promising regenerative and immunomodulatory properties in experimental settings; however, clinical evidence is limited, and existing studies are frequently constrained by small sample sizes, lack of appropriate controls, and short follow-up durations.

Variability in reported outcomes also reflects inconsistencies in study design, including differences in dosing strategies, treatment intervals, delivery platforms, and outcome assessment methodologies. In particular, the absence of standardized, objective endpoints for aesthetic improvement complicates comparison across studies and limits the ability to draw definitive conclusions regarding efficacy. Furthermore, the durability of treatment effects remains unclear, with some studies suggesting transient improvements without sustained structural remodeling.

Additional challenges arise from the lack of standardization and regulatory clarity surrounding several regenerative modalities. Variations in product composition, manufacturing processes, and characterization methods introduce substantial heterogeneity, raising concerns regarding reproducibility, safety, and quality control. These issues are particularly relevant for cell-derived products, where batch variability and incomplete characterization of bioactive components may influence biological activity and clinical outcomes.

Importantly, there is a marked absence of rigorously designed randomized controlled trials evaluating regenerative therapies for cosmetic indications in patients with diabetes. Given the distinct pathophysiological environment of diabetic skin, including impaired angiogenesis, chronic inflammation, and altered cellular responsiveness, therapeutic effects observed in non-diabetic populations may not be directly generalizable. Future investigations should prioritize standardized methodologies, well-defined clinical endpoints, and the inclusion of diabetic cohorts to establish evidence-based recommendations and clarify the role of regenerative therapies in this population.

### 6.6. Regulatory and Safety Considerations

Regulatory considerations represent an important and evolving aspect of regenerative therapies in aesthetic dermatology, particularly for mesenchymal stromal cell (MSC)–derived exosomes and secretomes. In contrast to platelet-rich plasma, which is generally classified as a minimally manipulated autologous product, MSC-derived biologics fall within a more complex regulatory landscape. Their classification varies across jurisdictions and may depend on factors such as degree of manipulation, source material, intended use, and route of administration, with some products categorized as biologics or advanced therapy medicinal products, while others are marketed under cosmetic frameworks.

At present, MSC-derived exosome and secretome therapies have not received widespread regulatory approval for aesthetic indications, and available products differ in terms of manufacturing processes, characterization methods, and composition. This variability may have implications for consistency and reproducibility across studies and clinical applications.

From a safety perspective, considerations include potential immunogenicity, variability in biological activity, and the limited availability of long-term clinical data. In addition, differences in dosing approaches and delivery protocols remain areas of ongoing investigation. Adverse event reporting in this field is still limited, particularly in non-standardized settings.

Overall, while these therapies show promise, further research and continued development of standardized manufacturing practices and regulatory frameworks will be important to support their safe and effective integration into clinical practice.

## 7. Proposed Best-Practices Framework for Aesthetic Care in Patients with Diabetes

**Figure 4:**
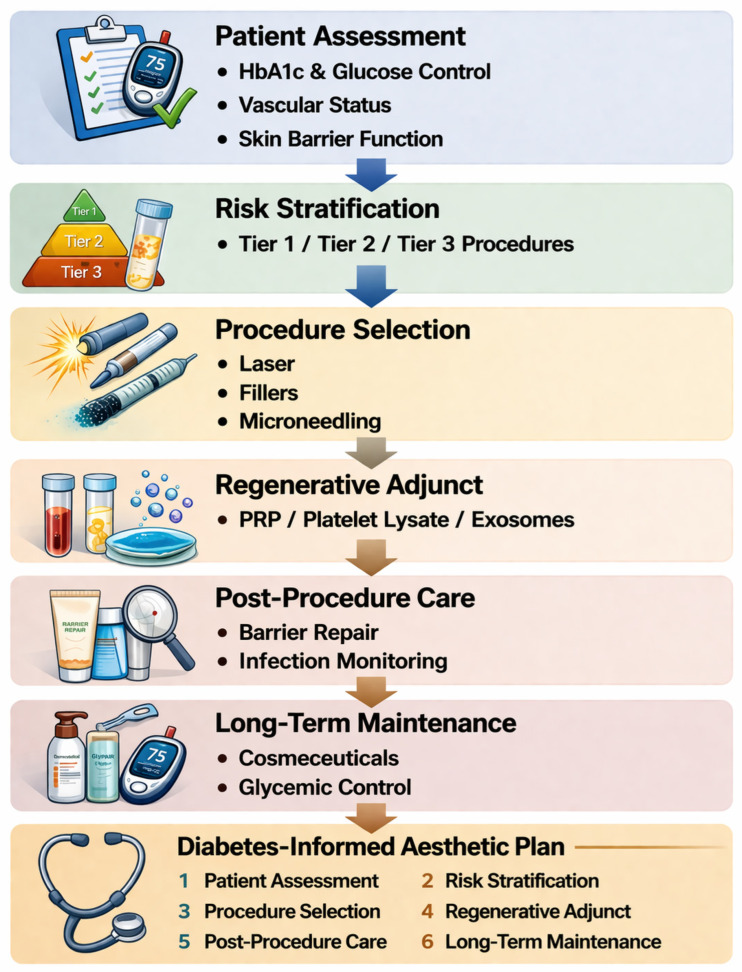
Diabetes-informed framework for aesthetic dermatologic management. Schematic representation of a structured clinical workflow for aesthetic procedures in patients with diabetes mellitus. Management begins with comprehensive patient assessment, including evaluation of glycemic control (HbA1c and glucose levels), vascular status, and epidermal barrier integrity. Patients are then risk stratified to guide appropriate procedure selection (e.g., laser therapies, dermal fillers, microneedling) according to metabolic stability and cutaneous risk factors. Regenerative adjuncts, including platelet-rich plasma (PRP), platelet lysate, or mesenchymal stromal cell–derived extracellular vesicles, may be incorporated to augment tissue repair and dermal remodeling. Post-procedural care emphasizes epidermal barrier restoration and surveillance for infection or delayed healing. Long-term maintenance integrates barrier-supportive cosmeceuticals and sustained glycemic optimization to support skin health and procedural outcomes. This framework outlines a risk-adapted, biology-informed approach to aesthetic care in diabetic patients. Created in BioRender. Mittal, R. (2026) https://BioRender.com/m61pjp4.

The following structured framework is proposed to guide clinicians in delivering safe and effective cosmetic care to patients with diabetes (Figure 4). It integrates principles from diabetic wound care, aesthetic dermatology, and regenerative medicine into a practical, stepwise approach (Table 4). Table 4 integrates key clinical considerations and proposed best practices for aesthetic management in patients with diabetes. This framework is intended to support clinical decision-making by aligning patient-specific factors, procedural selection, and post-procedural care with the underlying pathophysiology of diabetic skin.

### 7.1. Phase I: Pre-Procedural Assessment and Optimization

#### 7.1.1. Metabolic Assessment

All patients with diabetes seeking aesthetic procedures should undergo metabolic screening prior to treatment. Key parameters include: glycated hemoglobin (HbA1c)—ideally <7.0% for low-risk procedures, <8.0% as a ceiling for moderate-risk procedures, with higher-risk procedures deferred until glycemic optimization is achieved; fasting blood glucose and recent glucose monitoring trends; assessment of microvascular complications (neuropathy screening, peripheral vascular assessment); and renal function (eGFR) to inform drug metabolism and infection risk [84,169].

#### 7.1.2. Dermatologic Assessment

A comprehensive skin examination should evaluate: barrier function (clinical assessment of xerosis, TEWL measurement if available); skin elasticity and turgor (cutometry or clinical assessment); pigmentary status (Fitzpatrick phototype, presence of dyschromia, acanthosis nigricans); evidence of active infection, ulceration, or neuropathic changes; signs of microangiopathy (capillaroscopy, delayed capillary refill); and presence of diabetes-specific dermatoses that may influence treatment planning. Baseline photography with standardized lighting should be obtained [6,84].

#### 7.1.3. Barrier Optimization

Prior to any procedure that disrupts the epidermal barrier, a 2–4-week barrier optimization program is recommended: intensive moisturization with ceramide-containing or urea-based emollients (10–25% urea); correction of xerosis; treatment of any active infections; and consideration of topical antioxidants (vitamin C, niacinamide) to reduce baseline oxidative stress [28,170]. This preparatory phase improves the baseline skin condition, enhances tolerance of procedural stress, and may improve post-procedural recovery.

### 7.2. Phase II: Modality Selection

#### 7.2.1. Risk Stratification

Procedure selection should be guided by a diabetes-specific risk stratification that considers glycemic control status, presence of microvascular complications, immune status, and the invasiveness of the planned procedure. A tiered approach is recommended.

#### 7.2.2. Combining Regenerative and Conventional Approaches

A key principle of this framework is that regenerative therapies should be viewed as adjuncts to not replacements for conventional aesthetic procedures. The rationale for combining regenerative products with standard approaches is to compensate for the endogenous signaling deficits of diabetic skin. Practical examples include: PRP or platelet lysate applied topically during and immediately after microneedling to supply growth factors directly to the micro-channels; PRP injection preceding or concurrent with fractional laser therapy to enhance the neocollagenesis response; platelet lysate–enriched serums in post-procedural care regimens to support barrier recovery; and scalp PRP combined with microneedling for hair restoration, with consideration of supplemental topical growth factor products [91,101,102].

### 7.3. Phase III: Procedural Modifications

For patients with diabetes, several procedural modifications are recommended across aesthetic modalities.

Treatment intensity: Begin with conservative settings (lower laser fluences, shallower microneedling depths, milder peel concentrations) and titrate upward based on healing response. This approach prioritizes safety while allowing dose escalation in patients who demonstrate adequate healing capacity.

Treatment intervals: Extend inter-session intervals by 25–50% compared with standard protocols to allow for potentially delayed healing and collagen remodeling. For microneedling, consider 6–8-week intervals rather than the standard 4–6 weeks.

Infection prophylaxis: Maintain a lower threshold for prophylactic antibiotics (topical or systemic) in procedures that disrupt the epidermal barrier, particularly in patients with HbA1c > 7.5% or those with a history of skin infections. Antiviral prophylaxis should follow standard perioral guidelines.

Glycemic monitoring: For Tier 2 and Tier 3 procedures, blood glucose should be checked on the day of the procedure and post-procedurally if feasible. Hyperglycemia (>250 mg/dL) at the time of the procedure should prompt deferral of elective aesthetic treatments.

Aseptic technique: Enhanced aseptic technique is mandatory, reflecting the immunocompromised status of diabetic skin. This includes meticulous skin preparation, use of sterile single-use products, and avoidance of multi-dose vials.

### 7.4. Phase IV: Post-Procedural Care and Monitoring

Post-procedural management in diabetic patients should be more intensive than standard aesthetic aftercare:

Barrier recovery: Immediate application of ceramide-containing, fragrance-free barrier repair products. Avoidance of active ingredients (retinoids, alpha-hydroxy acids, vitamin C) for an extended period (7–14 days versus the standard 3–5 days) following procedures that disrupt the barrier.

Follow-up schedule: Earlier and more frequent follow-up than standard protocols, typically at 48–72 h post-procedure for Tier 2–3 procedures to assess healing trajectory and detect early signs of infection or delayed healing.

Patient education: Detailed post-procedural instructions emphasizing glucose monitoring, wound care, signs of infection, and when to seek urgent evaluation. Patients should be counseled that results may develop more slowly and require more treatment sessions than in non-diabetic patients.

Outcome assessment: Standardized photography at consistent intervals. Realistic expectation management is essential as patients with diabetes may achieve meaningful cosmetic improvement, but the magnitude of response to collagen-stimulating procedures may be attenuated compared with non-diabetic patients.

### 7.5. Phase V: Maintenance and Long-Term Skin Health

Long-term management should integrate aesthetic maintenance with diabetic skin health:

Daily skin care: Ceramide-based cleansers and moisturizers; daily broad-spectrum sunscreen (SPF ≥ 30) given the synergistic role of UV and AGEs in skin aging; topical antioxidants (vitamins C and E, niacinamide) to counteract oxidative stress; and consideration of topical anti-glycation agents (carnosine, aminoguanidine-containing formulations) as an emerging area of cosmeceutical research [39,171].

Glycemic optimization: Reinforcing the message that tight glycemic control is the single most effective anti-aging intervention for diabetic skin, as it slows AGE accumulation, reduces inflammation and oxidative stress, and preserves microvascular function [19,24].

Maintenance treatments: PRP or microneedling maintenance sessions at 6–12 month intervals, tailored to individual response and metabolic stability. These serve both cosmetic and skin health functions by periodically stimulating collagen remodeling in an otherwise stagnant dermal environment.

## 8. Anti-Glycation Strategies as Cosmetic Interventions

Given the central role of AGEs in diabetic skin aging, interventions that inhibit AGE formation, break existing AGE cross-links, or counteract AGE-mediated signaling represent a mechanistically targeted approach to cosmetic care in diabetes. Several categories of agents have been investigated [22,39,171].

AGE formation inhibitors include aminoguanidine, pyridoxamine (vitamin B6), benfotiamine (vitamin B1 analogue), and various botanical extracts (green tea catechins, carnosine, quercetin, and resveratrol). While some have shown efficacy in reducing skin collagen AGE accumulation in animal models, clinical data for topical dermatologic applications remain limited [22,39]. Notably, metformin, the most widely prescribed oral antidiabetic agent inhibits AGE formation through reactive carbonyl trapping and RAGE expression reduction providing a pharmacological link between glycemic management and anti-aging skin benefits that reinforces the centrality of metabolic optimization in the cosmetic care of diabetic patients [172,173,174,175].

AGE cross-link breakers such as alagebrium (ALT-711) have demonstrated the ability to cleave established AGE cross-links in vitro and in animal models, potentially restoring collagen flexibility [176]. However, clinical translation for dermatologic applications has been limited, and in vivo AGE cross-link breaking in human skin has not been convincingly demonstrated [177].

RAGE antagonists that block the AGE–RAGE signaling axis represent another therapeutic avenue, as they could attenuate the downstream inflammatory and oxidative cascades. This approach remains investigational.

Topical antioxidants (vitamin C, vitamin E, N-acetylcysteine, coenzyme Q10) can scavenge the ROS generated during AGE formation and RAGE activation, providing an indirect anti-glycation effect. These are the most readily available and best-studied agents for clinical use and can be recommended as adjunctive cosmeceuticals for patients with diabetes [171,178].

Integration of anti-glycation and antioxidant strategies into routine cosmeceutical care for patients with diabetes represents a low-risk, mechanistically sound approach, even in the absence of definitive clinical trial data from diabetic dermatologic cohorts. Their use should be complementary to glycemic optimization, which remains the most effective anti-glycation intervention.

## 9. Limitations

This review has some limitations. First, as a narrative review, it does not employ systematic search methodology and may be subject to selection bias. Second, the evidence base for aesthetic procedures and regenerative therapies in diabetic patients is remarkably thin most aesthetic studies exclude diabetic patients, and most regenerative studies focus on wound healing rather than cosmetic endpoints. The best practices framework proposed here is therefore largely expert opinion based and informed by extrapolation from related evidence domains rather than derived from high quality diabetic specific aesthetic trials. Third, the heterogeneity of diabetes encompassing type 1 and type 2 disease, varying durations, glycemic control levels, and complication profiles makes generalizations difficult. The distinct autoimmune pathophysiology of type 1 diabetes may confer different skin aging patterns and procedural risks compared with the metabolic syndrome driven pathology of type 2 diabetes, yet existing literature rarely stratifies outcomes by diabetes type. Fourth, the regenerative therapy field is rapidly evolving, and the evidence summarized here may be superseded. Fifth, patient-reported outcome data for cosmetic satisfaction in diabetic populations are virtually absent. Future research should prioritize well-designed clinical trials evaluating aesthetic procedures and regenerative therapies specifically in diabetic cohorts, with both objective outcome measures and patient reported endpoints.

## 10. Conclusions and Future Directions

Patients with diabetes represent a large, growing, and underserved population in cosmetic dermatology. Their skin undergoes accelerated aging through mechanisms that are mechanistically distinct from chronological and photoaging, driven primarily by AGE accumulation, chronic inflammation, oxidative stress, microangiopathy, and neuropathy. These processes alter the safety and efficacy of conventional aesthetic procedures and create a compelling rationale for regenerative approaches that target the underlying biological deficits.

Among regenerative therapies, PRP has the most established though still limited evidence for cosmetic applications and is readily available for clinical use. Platelet lysate offers theoretical advantages in bypassing patient-specific platelet dysfunction but requires further clinical validation. MSC-derived exosomes and secretomes demonstrate exciting preclinical potential but remain far from routine clinical application, and the unregulated commercial marketing of these products raises patient safety concerns.

Despite increasing interest in MSC-derived exosomes and secretomes as regenerative strategies in aesthetic dermatology, their clinical application remains investigational. The current data is predominantly derived from preclinical studies and early-phase clinical investigations, with limited high-quality, controlled data to support efficacy and safety, particularly in patients with diabetes mellitus. Critical challenges persist, including lack of standardization in isolation and characterization methods, variability in bioactive cargo, and insufficient long-term safety data. Accordingly, these biologic therapies should not be considered part of routine clinical practice at present, and their use should be confined to rigorously designed clinical trials until further evidence becomes available.

The structured best-practices framework proposed in this review, encompassing metabolic and dermatologic pre-assessment, barrier optimization, risk-stratified modality selection, procedural modifications, enhanced post-procedural care, and long-term maintenance, provides a practical starting point for clinicians serving diabetic patients. This framework should be refined as evidence accumulates.

Future priorities include: (a) clinical trials evaluating aesthetic outcomes (skin texture, elasticity, pigmentation, patient satisfaction) of standard and regenerative procedures in diabetic versus non-diabetic cohorts; (b) validation of diabetes-specific risk stratification criteria; (c) development of anti-glycation cosmeceuticals with demonstrated efficacy in diabetic skin; (d) standardization of PRP preparation and regenerative product manufacturing for aesthetic applications; (e) long-term safety monitoring for regenerative products in immunocompromised patients; (f) incorporation of patient-reported outcome measures in diabetic aesthetic research; and (g) investigation of emerging systemic therapies, including glucagon-like peptide-1 receptor agonists and sodium-glucose cotransporter-2 inhibitors, for potential secondary benefits on skin aging biomarkers through improved glycemic control, anti-inflammatory effects, and metabolic modulation.

Ultimately, patients with diabetes deserve the same access to evidence-based, safe, and effective aesthetic care as the general population. Meeting this standard requires recognizing diabetic skin as a distinct biological entity and adapting our aesthetic approaches accordingly.

## Figures and Tables

**Table 1:** Comparative features of chronological aging, photoaging, and diabetes-accelerated skin aging.

Feature	Chronological Aging	Photoaging	Diabetic Skin Aging
Primary driver	Time/genetics [16,17]	UV radiation [18]	Chronic hyperglycemia, and persistent inflammatory signaling [19]
AGE accumulation	Slow, linear with age [20]	Moderate (UV-accelerated) [21]	Rapid, severalfold above age-matched controls [22]
Collagen changes	Gradual loss (~1%/year); normal turnover [17]	MMP-mediated degradation; solar elastosis [23]	Cross-linking and impaired turnover; stiffening; MMP resistance [22]
Elastin	Slow fragmentation [16]	Elastotic degeneration (thickened, tangled) [18]	AGE cross-linking; thinned, rigid, non-functional [24]
Inflammation	Mild inflammaging [17]	UV-induced; episodic [25]	Chronic, systemic; M1 macrophage skew [26]
Oxidative stress	Gradual mitochondrial decline [20]	UV-generated ROS [23]	Polyol pathway, PKC, AGE–RAGE; severe [22]
Vasculature	Gradual capillary loss [27]	Telangiectasia; actinically damaged vessels [18]	Microangiopathy; basement membrane thickening; endothelial dysfunction [28]
Barrier function	Mild decline in lipid synthesis [17]	Variable; UV damage to lipid lamellae [21]	Reduced ceramides, cholesterol; increased TEWL; severe xerosis [29]
Pigmentation	Melanocyte loss (pallor) [16]	Lentigines; mottled dyschromia [25]	AGE-driven yellowing; RAGE-mediated melanogenesis; acanthosis nigricans [22]
Neurological	Mild sensory decline [27]	Not primary feature [18]	Peripheral neuropathy; reduced neuropeptide signaling [26]
Healing capacity	Slowed but functional [17]	Mildly impaired in severely photodamaged skin [25]	Significantly impaired; delayed re-epithelialization; infection risk [26]
Clinical appearance	Fine wrinkles; pallor; mild laxity [16]	Coarse wrinkles; leathery texture; lentigines [18,25]	Premature aging; sallow/yellow tone; severe xerosis; skin tags; dermopathy [30]
Cosmetic procedure risk	Standard age-related considerations [17]	Increased sensitivity to resurfacing [23]	Elevated: delayed healing, infection, dyschromia, reduced efficacy [30]

Abbreviations: UV, ultraviolet; AGE, advanced glycation end product; MMP, matrix metalloproteinase; ROS, reactive oxygen species; PKC, protein kinase C; RAGE, receptor for advanced glycation end products; TEWL, transepidermal water loss.

**Table 2:** Aesthetic procedure safety considerations in patients with diabetes mellitus.

Procedure	Risk Level in Diabetes	Primary Concerns	Evidence Type	Evidence Level	Key Modifications Needed
Neuromodulators	Low	Altered neuromuscular sensitivity; bruising	Indirect clinical	Very limited	Monitor duration of effect; gentle injection technique
HA fillers	Low–Moderate	Infection; biofilm; altered longevity	Indirect clinical	Limited	Strict asepsis; glycemic optimization; avoid immunosuppressed patients
Biostimulatory fillers	Moderate	Altered neocollagenesis; unpredictable results	Mechanistic + indirect clinical	Very limited	Consider reduced expectations; close follow-up
Superficial peels	Low	Barrier disruption; irritation	Indirect clinical	Limited	Barrier-supportive aftercare; avoid in active infection
Medium peels	Moderate	Delayed healing; dyschromia; infection	Indirect clinical + diabetic wound-healing extrapolation	Very limited	HbA1c <8%; prophylactic measures; extended healing protocol
Deep peels	High	Non-healing; scarring; systemic absorption	Mechanistic + diabetic wound-healing data	Very limited	Generally contraindicated in poorly controlled diabetes
Non-ablative fractional laser	Low–Moderate	Reduced collagen response; delayed recovery	Indirect clinical	Limited	Conservative settings; extended intervals between sessions
Ablative fractional laser	Moderate–High	Delayed healing; infection; scarring	Indirect clinical + diabetic wound-healing extrapolation	Very limited	HbA1c optimization; prophylaxis; modified parameters
Microneedling	Low–Moderate	Reduced collagen induction; infection	Indirect clinical + mechanistic	Very limited	Conservative depth; combine with PRP; infection prophylaxis
PRP (face/scalp)	Low	Reduced autologous potency; injection site healing	Indirect clinical + mechanistic	Limited	Consider platelet function; combine with microneedling

Abbreviations: HA, hyaluronic acid; HbA1c, glycated hemoglobin; PRP, platelet-rich plasma. Evidence Type Definitions: Direct clinical = studies in patients with diabetes undergoing aesthetic procedures; Indirect clinical = studies in non-diabetic populations; Mechanistic/preclinical = in vitro, animal, or biological rationale studies.

**Table 3:** Regenerative therapy modalities for cosmetic applications in diabetic patients.

Feature	PRP	Platelet Lysate	MSC Exosomes	MSC Secretome
Source	Autologous blood	Autologous or allogeneic	Allogeneic MSC culture	Allogeneic MSC culture
Evidence Type	Indirect clinical + mechanistic	Mechanistic	Preclinical	Preclinical
Evidence Level	Moderate (general population); absent diabetic cosmetic data	Very limited	Very limited	Very limited
Cosmetic applications	Hair restoration; facial rejuvenation; scar; microneedling adjunct	Topical/injectable growth factor source; microneedling adjunct	Topical adjunct to microneedling/laser; cosmeceutical formulations	Topical/injectable; microneedling adjunct; comprehensive skin restoration
Diabetes advantage	Autologous; no immunogenicity	Bypasses patient platelet dysfunction; standardizable	Targetable cargo; anti-inflammatory	Multimodal signaling; addresses multiple deficits
Diabetes concern	Reduced potency from platelet dysfunction	Limited clinical evidence	Unregulated market; limited evidence	Batch variability; limited evidence
Clinical evidence for cosmetic use	Moderate (general pop.); absent in diabetic cosmetic	Very limited	Very limited; mostly preclinical	Very limited; mostly preclinical
Regulatory status	Medical device (PRP kits)	Biologic	Uncertain; evolving	Biologic
References	[22,92,93,94]	[95,96]	[97,98,99]	[100]

Abbreviations: PRP, platelet-rich plasma; MSC, mesenchymal stromal cell. Evidence Type Definitions: Direct clinical = studies in patients with diabetes undergoing aesthetic procedures; Indirect clinical = studies in non-diabetic populations; Mechanistic/preclinical = in vitro, animal, or biological rationale studies.

**Table 4:** Clinical framework for aesthetic dermatologic management in patients with diabetes mellitus.

Clinical Domain	Key Considerations	Evidence Type	Evidence Level (Quality)	Practical Recommendations	References
Patient selection	Assess glycemic control, comorbidities, infection risk	Indirect clinical + diabetic wound-healing extrapolation	Limited	Defer elective procedures if poorly controlled diabetes	[22,26,162]
Glycemic optimization	HbA1c threshold and metabolic stability	Indirect clinical + diabetic wound-healing extrapolation	Limited	Aim for HbA1c <8% prior to procedures	[26,163]
Skin assessment	Evaluate xerosis, dyschromia, barrier integrity	Indirect clinical + mechanistic	Limited	Treat xerosis and barrier dysfunction before procedures	[29]
Procedure selection	Match procedure invasiveness to patient risk profile	Indirect clinical	Limited	Prefer non-invasive or superficial procedures initially	[164]
Infection prevention	Increased susceptibility to infection and delayed healing	Indirect clinical + diabetic wound-healing extrapolation	Limited	Strict aseptic technique; consider prophylaxis in high-risk cases	[26]
Healing monitoring	Delayed re-epithelialization and complication risk	Indirect clinical + mechanistic	Limited	Extend follow-up intervals and monitor closely	[165]
Use of regenerative adjuncts	PRP, growth factors, MSC-based therapies	Indirect clinical + mechanistic	Very limited	Consider as adjuncts to enhance healing	[124,166]
Patient counseling	Set realistic expectations and risk communication	Indirect clinical	Limited	Discuss delayed healing and variable outcomes	[167,168]

Abbreviations: HbA1c, glycated hemoglobin; HA, hyaluronic acid; PRP, platelet-rich plasma; IPL, intense pulsed light; LED, light-emitting diode. Evidence Type Definitions: Direct clinical = studies in patients with diabetes undergoing aesthetic procedures; Indirect clinical = studies in non-diabetic populations; Mechanistic/preclinical = in vitro, animal, or biological rationale studies.

## Data Availability

No new data were created or analyzed in this study. Data sharing is not applicable to this article.

## Abbreviations

AGE	Advanced glycation end products
CGRP	Calcitonin gene-related peptide
CREB	cAMP response element-binding protein
EGF	Epidermal growth factor
ERK	Extracellular signal-regulated kinase
HA	Hyaluronic acid
HbA1c	Glycated hemoglobin
IL	Interleukin
IPL	Intense pulsed light
KTP	Potassium titanyl phosphate
MMP	Matrix metalloproteinase
MSC	Mesenchymal stromal cell
NF-Κb	Nuclear factor kappa B
PDGF	Platelet-derived growth factor
PKC	Protein kinase C
PRP	Platelet-rich plasma
RAGE	Receptor for advanced glycation end products
ROS	Reactive oxygen species
TEWL	Transepidermal water loss
TGF-β	Transforming growth factor beta
TNF-α	Tumor necrosis factor alpha
UV	Ultraviolet
VEGF	Vascular endothelial growth factor
